# 1,8-Cineole Ameliorates Steatosis of Pten Liver Specific KO Mice via Akt Inactivation

**DOI:** 10.3390/ijms160612051

**Published:** 2015-05-27

**Authors:** Soichiro Murata, Koichi Ogawa, Takashi Matsuzaka, Mitsuru Chiba, Ken Nakayama, Kenichi Iwasaki, Tomohiro Kurokawa, Naoki Sano, Tomohito Tanoi, Nobuhiro Ohkohchi

**Affiliations:** 1 Department of Surgery, Faculty of Medicine, University of Tsukuba, 1-1-1 Tennodai, Tsukuba, Ibaraki 305-8575, Japan; E-Mails: k-ogawa@md.tsukuba.ac.jp (K.O.); kenken.nakayama@gmail.com (K.N.); pinpon1225@yahoo.co.jp (K.I.); yuuhaku@gmail.com (T.K.); sanuuuh19@gmail.com (N.S.); tomohito316@hotmail.com (T.T.); nokochi3@md.tsukuba.ac.jp (N.O.); 2 Department of Endocrinology and Metabolism, Faculty of Medicine, University of Tsukuba, 1-1-1 Tennodai, Tsukuba, Ibaraki 305-8575, Japan; E-Mail: t-matsuz@md.tsukuba.ac.jp; 3 Department of Biomedical Sciences, Division of Medical Life Sciences, Graduate School of Health Sciences, Hirosaki University, Hirosaki, Aomori 036-8564, Japan; E-Mail: mchiba32@cc.hirosaki-u.ac.jp

**Keywords:** 1,8-cineole, NASH, PTEN, Akt, LXR alpha

## Abstract

Hepatocyte-specific Phosphatase and tensin homolog (Pten)-knockout (KO) mice exhibit hepatic lesions analogous to non-alcoholic steatohepatitis (NASH). 1,8-cineole is a monoterpene oxide and it has several biological effects including hepatoprotective effects. In this study we revealed that 1,8-cineole ameliorates NASH of Pten KO mice. Pten KO mice were assigned to a control group without any medication or to a 1,8-cineole group injected with 50 mg/kg i.p. twice per week for eight weeks. At eight weeks, livers from each group were processed to measure triglyceride (TG) content, gene expression analysis, western blot analysis, and histological examination including Oil red O staining. 1,8-cineole ameliorated hepatic steatosis in Pten KO mice, revealed by TG content and Oil red O staining. Moreover, 1,8-cineole downregulated collagen 1a1 expression and improved liver fibrosis. Thus, 1,8-cineole has potential as a candidate to treat NASH by inactivating the Akt/PI3-kinase pathway.

## 1. Introduction

Non-alcoholic fatty liver disease (NAFLD) is characterized by triglyceride (TG) accumulation in hepatocytes without chronic alcohol consumption. NAFLD is the most common cause of liver dysfunction [1]. Non-alcoholic steatohepatitis (NASH) is a severe form of NAFLD, which is accompanied by cellular damage, inflammation, and fibrosis. NASH is becoming a serious public health problem worldwide. NASH often progresses to liver cirrhosis and even hepatocellular carcinoma (HCC) [2].

The two-hit hypothesis has been proposed to explain NASH [3]. The first hit is hepatic lipid accumulation and the second hit is oxidative stress, proinflammatory cytokines, and adipocytokines, which induce necroinflammation in the liver [2]. The precise mechanism of the first hit is still unclear. One of the candidates of the first hit is overactivation of Akt/PI3-kinase. Activation of PI3-kinase generates 3-phospholipids including phosphatidylinositol 3,4,5-triphosphate, which mediates many cellular responses to insulin. Phosphatase and tensin homolog (Pten) attenuates phosphatidylinositol 3,4,5-triphosphate level [4]. Horie *et al.* established hepatocyte-specific Pten-deficient (Pten KO) mice to investigate the role of Pten in the liver [5]. These Pten KO mice were reported to have steatohepatitis with ballooned hepatocytes, Mallory’s hyaline, lobular inflammation and pericellular fibrosis [5]. In the liver of Pten KO mice, production of fatty acids to the hepatocytes is increased by the enhanced expression of fatty acid synthase (FASN) and Sterol regulatory element-binding protein 1c (SREBP-1c), and these molecules are downstream of Akt/PI3-kinase. Pten KO mice not only spontaneously develop steatohepatitis and hepatic fibrosis but also hepatocellular carcinoma and these mice resemble the natural history of NASH [5].

Several essential oils from plants possess medical benefits. Among the various constituents of essential oils, 1,8-cineole has been shown to possess pharmacological effects. The content of 1,8-cineole in the essential oils varies in the different Eucalyptus species, from 25% to 90% [6,7]. 1,8-cineole has been used as a percutaneous penetration enhancer [8], an antibacterial and expectorant [6], and as an anti-inflammatory [9,10] or antihypertensive [11] agent. We revealed that 1,8-cineole has an anti-cancer effect against colorectal cancer [12]. Although there is a report about 1,8-cineole and fatty liver in zebrafish [13], the effect of 1,8-cineole against fatty liver in mammals is still unclear. The purpose of this study is to evaluate the ameliorative effect of 1,8-cineole against NASH of Pten KO mice.

## 2. Results and Discussion

### 2.1. Results

#### 2.1.1. 1,8-Cineole Ameliorates NASH in Pten KO Mice

**Figure 1:**
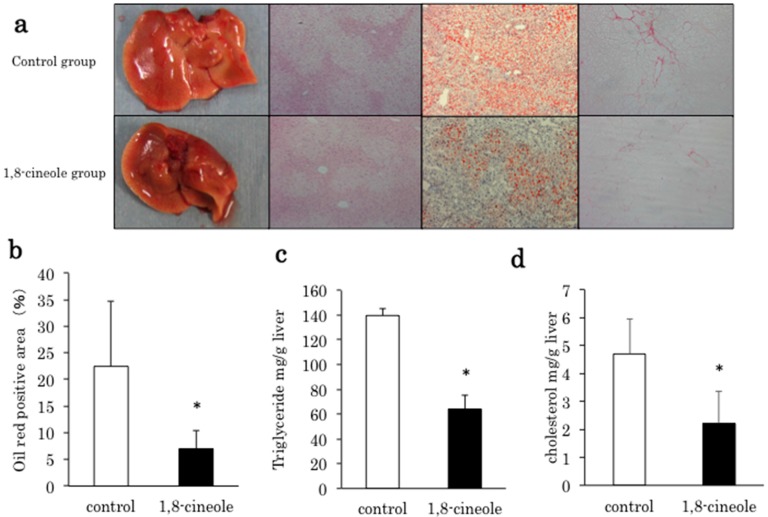
(**a**) Macroscopic and microscopic views of liver of 20-week-old Pten KO mice without any treatment (**upper**; control) and 1,8-cineole treatment for eight weeks (**lower**; 1,8-cineole). The liver in the control group (first line upper) was enlarged, where as the liver in the 1,8-cineole group was smaller than that in the control group. HE stained livers of the control group (100× second line upper) and the 1,8-cineole group (100× second line lower) indicate vacuoles in hepatocytes were decreased in the 1,8-cineole group. Lipid accumulation was confirmed by Oil red O staining (100× control; third upper, 1,8-cineole group; third lower). Sirius red staining of the liver (400× control; fourth upper, 1,8-cineole; fourth lower). In the 1,8-cineole group, fibrotic area is narrower than in the control group; (**b**) Oil red O positive area (%) is compared between the two groups. *, *p* < 0.05 *vs*. control group. Control = 4, 1,8-cineole = 5; (**c**) Hepatic triglyceride content. Control = 5, 1,8-cineole = 6; (**d**) Hepatic cholesterol content. *n* = 3 in each group; content was significantly lower in the 1,8-cineole group compared to the control group. * *p* < 0.05 *vs.* control.

Eight-week continuous intraperitoneal injection of 1,8-cineole ameliorated lipid accumulation of livers compared to the control group. The body weight, liver weight, liver/body weight %, and serum parameters are listed on Table 1. No significant weight loss was observed between the two groups. Liver weight was significantly lower in the 1,8-cineole group. Serum cholesterol of the 1,8-cineole group was significantly decreased compared to the control group. Serum insulin was significantly increased in the 1,8-cineole group. Figure 1a shows gross appearance of liver, HE staining, Oil red O staining, and Sirius red staining. In the 1,8-cineole group, lipid droplet and fibrotic area were decreased compared to the control group. Figure 1b shows the Oil red O positive area and that of the 1,8-cineole group was significantly lower than the control group. Figure 1c shows triglyceride content and Figure 1d shows cholesterol content of the liver. Both triglyceride and cholesterol contents were significantly decreased in the 1,8-cineole group compared to the control group.

**Table 1:** Body, liver weight, and serum biochemical analyses of control and 1,8-cineole.

Parameters	Control	1,8-Cineole
**Weight**		
Body weight (g)	32.40 ± 2.20	31.11 ± 1.37
Liver weight (g)	3.25 ± 0.40	2.83 ± 0.44 ^A^
Liver/Body weight (%)	10.06 ± 0.98	9.10 ± 1.41
**Serum**		
ALT (IU/L)	172.6 ± 91.9	245.1 ± 90.2
Triglyceride (mg/dL)	89.6 ± 26.4	95 ± 68.2
Total cholesterol (mg/dL)	116.6 ± 26.9	85.5 ± 15 ^A^
Glucose (mg/dL)	144.6 ± 39.4	150.4 ± 30.2
Insulin (mU/L)	0.24 ± 0.18	0.61 ± 0.33 ^A^

Results are expressed as the mean ± SD. Statistical difference was determined using Mann-Whitney’s *U* test. ^A^ *p* < 0.05 *vs*. control. Control = 6; 1,8-cineole = 8.

#### 2.1.2. Signal Induction in the Liver

**Figure 2:**
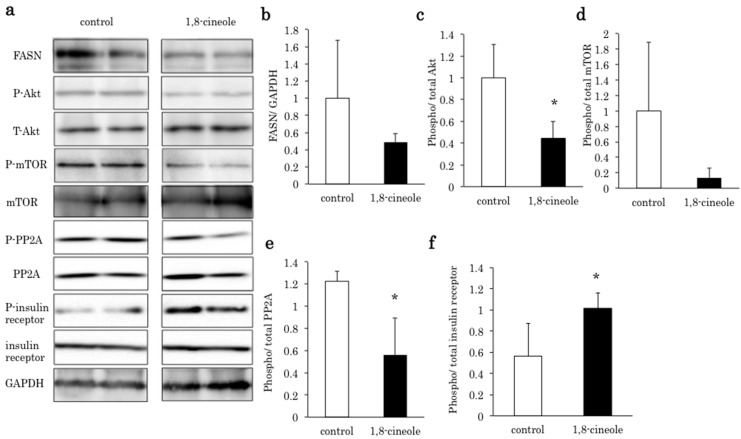
(**a**) Western blot of fatty acid synthase (FASN), phospho Akt (P-Akt), total Akt (T-Akt), phospho mTOR (P-mTOR), total mTOR (mTOR), Phospho-protein phosphatase type 2A (P-PP2A), total PP2A (PP2A), phospho-insulin receptor (P-insulin receptor), total insulin receptor (insulin receptor) and Glyceraldehyde-3-phosphate dehydrogenase (GAPDH). In the 1,8-cineole group, FASN, P-Akt, P-mTOR, and P-PP2A were decreased. P-insulin receptor was increased in the 1,8-cineole group. Densitometry of FASN/GAPDH (**b**), phospho/total Akt (**c**), phospho/total mTOR (**d**), phospho/total PP2A (**e**), and phospho/total insulin receptor (**f**) in the two groups (*n* = 3). In the 1,8-cineole group, Akt and PP2A were significantly dephosphorylated, and the insulin receptor was significantly activated. * *p* < 0.05 *vs*. control.

Figure 2a shows a western blot of the liver of the control and 1,8-cineole groups. In the 1,8-cineole group, FASN, phospho Akt, phospho mTOR were decreased compared to the control. The phospho-insulin receptor was strongly activated in the 1,8-cineole group. Densitometry of FASN/GAPDH (Figure 2b), phospho/total Akt (Figure 2c), phospho/total mTOR (Figure 2d), phospho/total PP2A (Figure 2e), and phospho/total insulin receptor (Figure 2f) in the two groups are shown. In the 1,8-cineole group, Akt and PP2A were significantly dephosphorylated, and the insulin receptor was significantly activated compared to the control group. FASN and phospho mTOR expression were also decreased in the 1,8-cineole group without significant difference.

#### 2.1.3. Gene Expression of the Liver

**Figure 3:**
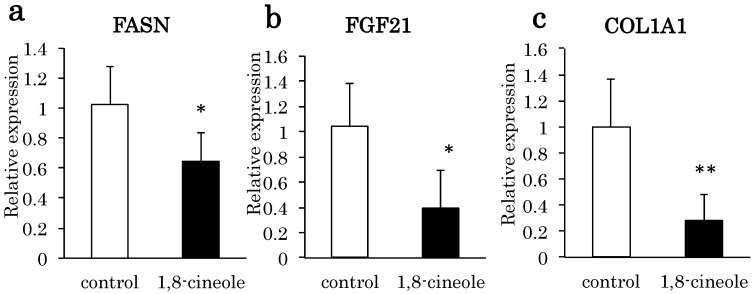

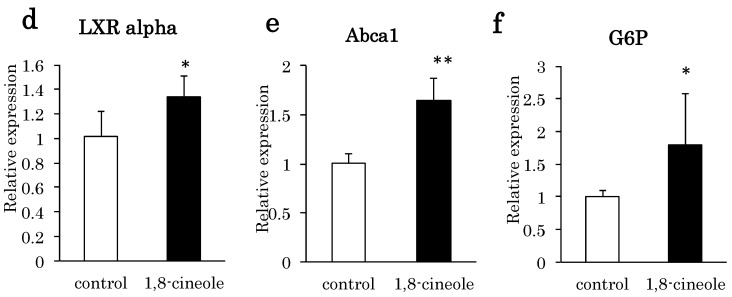
RT-PCR of the liver messenger RNA in the two groups. (**a**) fatty acid synthase (FASN); (**b**) fibroblast growth factor 21 (FGF21); (**c**) collagen 1A1 (COL1A1); (**d**) Liver X Receptor alpha (LXR alpha); (**e**) ATP-binding cassette, sub-family A, member 1 (Abca1); and (**f**) Glucose 6 Phosphatase (G6P). Data shows relative expression normalized to Glyceraldehyde-3-phosphate dehydrogenase (GAPDH). * *p* < 0.05, ** *p* < 0.01 *vs*. control. *n* = 3.

By RT-PCR analysis, FASN, FGF21, and COL1A1 expression were decreased significantly in the 1,8-cineole group compared to the control group (Figure 3a–c). LXR alpha and downstream Abca1 were upregulated significantly compared to the control group (Figure 3d,e). As insulin concentration was restored in the 1,8-cineole group, G6P was upregulated compared to the control group (Figure 3f).

### 2.2. Discussion

In this study, we demonstrated that 1,8-cineole administration improved steatosis and fibrosis seen in Pten KO mice. We confirmed the decreased hepatic TG and cholesterol accumulation in these mice by lipid analysis. 1,8-cineole treatment is involved in many pathways and genes related to synthesis of lipid and hepatic fibrosis confirmed by western blot and RT-PCR.

Hyperactivation of Akt is the main reason of steatosis, fibrosis, and carcinogenesis of Pten KO mice. By activating PP2A, LXR alpha and downstream Abca 1, 1,8-cineole inactivated Akt and finally reduced steatosis and fibrosis of the Pten KO mice. Pten is a tumor suppressor gene which inactivates Akt. Hyperactivation of Akt causes steatosis, fibrosis and carcinogenesis [4]. Induced activation of Akt also causes steatosis in the murine liver [14]. In clinical study, mutations of Pten cause insulin hypersensitivity and obesity and the insulin hypersensitivity could be explained by the presence of enhanced insulin signaling through the PI3K-Akt pathway, as evidenced by increased Akt phosphorylation [15]. In our study, insulin concentration in the control group was lower than that in the 1,8-cineole group. Serum glucose levels were almost equal between the two groups. That means 1,8-cineole reduced the insulin hypersensitivity of Pten KO mice. As a result, G6P expression and phospho insulin receptor were activated in the 1,8-cineole group. In a previous study, we found that 1,8-cineole inactivated Akt in the human colorectal cancer cell line RKO [12]. Akt activation is tightly controlled by counteraction of PTEN, PP2A, and pleckstrin homology domain leucine-rich repeat protein phosphatase (PHLPP) [16]. It has long been known that PP2A negatively regulates Akt activity in various systems [16]. In this study, dephosphorylation of PP2A, which means activation of PP2A, causes inactivation of Akt and downstream mTOR, as observed in the 1,8-cineole group.

LXR alpha activation upregulates Abca1 and Abcg1. These molecules inactivate Akt in the lipid raft of the cellular membrane [17]. T0901317 is an agonist of LXR alpha. By inactivating Akt in the prostate cancer cell line, T0901317 is expected to be a chemotherapeutic agent [17]. Activation of LXR results in increased HDL levels and net whole body cholesterol loss [18]. There is a report that LXR activation by T0901317 exacerbated steatosis in high fat diet treated mice [19]. 1,8-cineole is reported to activate LXR alpha, Abca1, and Abcg1 in the murine macrophage cell line RAW 246.7 [20]. In our study, expression of LXR alpha and downstream Abca1 was elevated in 1,8-cineole mouse liver. One of the reasons for Akt inactivation by 1,8-cineole would be due to LXR alpha activation, but the relationship between LXR alpha and steatosis is still controversial.

FGF21 induces hepatic fatty acid oxidation by transcriptional regulation of key enzymes of fatty acid oxidation [21]. Dasarathy *et al.* revealed that plasma FGF21 concentration was higher in NASH than in controls [22]. FGF21 is also activated by hepatocarcinogenesis and hepatic stress [23]. FGF21 is an Akt dependent myokine [24], and LXR alpha activation is reported to reduce FGF 21 [25]. In our study, FGF 21 expression was decreased in the 1,8-cineole group, a result of Akt inactivation and LXR alpha activation.

In the NASH liver, fibrosis progresses and liver cirrhosis occurs [2]. There is a clinical evidence that hepatic Akt expression correlates with advanced fibrosis in patients with chronic hepatitis C infection [26]. Higher expression of Akt exacerbates liver fibrosis. 1,8-cineole treatment decreased fibrotic area stained by Sirius red in the liver, and COL1A1 expression was decreased.

1,8-cineole was reported to have various pharmacological effects, such as smooth muscle relaxant, anti-inflammation, antioxidant and hypotension [6,7,8,9,10,11]. Hepatoprotection was suggested to be associated with a reduction in TNF-α serum concentration [27]. Lima *et al.* reported that 1,8-cineole ameliorates cerulein-induced acute pancreatitis in mice by oral administration of 1,8-cineole from 100 to 400 mg/kg [28]. The effects of acute and subacute toxicity of 1,8-cineole in Kunming mice were studied. After acute oral administration, the LD_50_ value (95% Cl) was 3849 mg/kg. In the subacute toxicity study, there were no significant differences in body weight and relative organ weight between the control group and the 1,8-cineole treatment groups for 30 days with up to 192.45 mg/kg oral administration [29]. Although there are few reports on i.p. administration, 50 mg/kg of 1,8-cineole is assumed to be safe. Essential oil of Rosemary, which contains about 40% 1,8-cineole, was reported to have a hepatoprotective effect against CCl4 [30]. In our study, 1,8-cineole not only ameliorates steatosis, but also reduced fibrosis in Pten KO mice.

## 3. Experimental Section

### 3.1. Reagents

1,8-cineole(eucalyptol) was obtained from Sigma (St. Louis, MO, USA). For cell culture and i.p. injections, 1,8-cineole was dissolved in ethanol and diluted into water at a concentration of 10 mg⁄mL. In *in vivo* experiments, i.p. treatment with 1,8-cineole at 50 mg/kg was given twice a week for eight weeks.

### 3.2. Animals

Pten flox/flox mice (129Ola_C57BL6/J F2), generated as previously described [31], were mated to AlbCre transgenic mice (C57BL6/J background; The Jackson Laboratory, Bar Harbor, ME, USA) [32] in which expression of Cre is controlled by the promoter of the hepatocyte-specific gene Albumin. Offspring carrying AlbCre and two copies of the floxed Pten allele (AlbCrePtenflox/flox) were used in this study as homozygous mutant (Pten KO) mice [33]. Animal experiments were carried out in a humane manner after receiving approval from the Institutional Animal Experiment Committee of the University of Tsukuba (identification code 14-377, 2 September 2014) and in accordance with the Regulation for Animal Experiments at our university and the Fundamental Guidelines for Proper Conduct of Animal Experiments and Related Activities in Academic Research Institutions under the jurisdiction of the Ministry of Education, Culture, Sports, Science and Technology of Japan.

### 3.3. Treatment

Twelve-week-old male Pten KO mice were randomly divided into two groups: control group, mice without any treatment; and the 1,8-cineole group, mice with 50 mg/kg of 1,8-cineole administered twice a week i.p. for eight weeks. In each group, six to eight mice were used. At 20 weeks of age, the mice were sacrificed and specimens collected.

### 3.4. Histology and Oil Red O Staining

Formalin-fixed tissues were embedded in paraffin using standard procedures. Sections (4 μm thick) were cut and stained with either hematoxylin-eosin (HE) for standard microscopy or Sirius red stain to show fibrosis. To visualize lipids, frozen sections (5 μm thick) were stained with Oil Red O (Nakarai Tesque Inc., Kyoto, Japan) and counterstained with hematoxylin.

### 3.5. Biochemical Analyses of Liver Extracts and Serum

Levels of triglyceride (TG) (Abnova, Taipei, Taiwan) and cholesterol (Wako Pure Chemical Industries, Osaka, Japan) in total lipids extract of the liver and alanine aminotransferase (ALT), TG, cholesterol, glucose (Fuji Film, Tokyo, Japan), and insulin (Mercodia, Uppsala, Sweden) were determined by colorimetric, or enzymatic assays.

### 3.6. Western Blot Analysis

For western blot analysis, total protein extracts of control and 1,8-cineole group mice livers were obtained, then separated by 10% SDS-PAGE and transferred to nitrocellulose membrane (Millipore, Bedford, MA, USA). The following antibodies were used as primary antibodies: total Akt, phosphoserine 473 Akt, phospho and total mammalian target of rapamycin (mTOR) and Glyceraldehyde-3-phosphate dehydrogenase (GAPDH) (Cell Signaling Technology, Beverly, MA, USA). Purified mouse anti-FASN antibody was purchased from BD Biosciences (San Jose, CA, USA). Phospho (Tyr 972) and total insulin receptor were purchased from Merck Millipore (Darmstadt, Germany). Phospho and total PP2A were purchased from Abcam (Cambridge, MA, USA). Secondary goat anti rabbit and goat anti mouse antibodies conjugated with horseradish peroxidase were purchased from Cell Signaling Technology. Immunoblots were analyzed by enhanced chemiluminescence. Densitometry of phospho/total Akt was calculated by Image J [34].

### 3.7. Total RNA Extraction

For reverse transcription-polymerase chain reaction (RT-PCR), total RNA was extracted from 50 mg liver samples in *in vitro* and *in vivo* experiments. Total RNA was isolated from whole cells using a NucleoSpin^®^ RNA II kit (Macherey-Nagel, Düren, Germany) according to the manufacturer’s instructions. RNA concentrations were determined by measuring the absorbance at 260/280 nm with a NanoDrop Spectrophotometer (NanoDrop Technologies, Wilmington, DE, USA). The synthesis of complementary DNA was performed using AMV reverse transcriptase (Promega, Madison, WI, USA) and random primers (Takara Bio Inc., Shiga, Japan). Briefly, a mixture of 1 mM dNTPs (Fermentas Life Sciences, Burlingame, ON, Canada), 0.025 μg/mL random primers, 0.25 U/mL reverse transcriptase, and 500 ng of total RNA was incubated at 30 °C for 10 min, 37 °C for 60 min, 95 °C for 5 min and at 4 °C before storage at −80 °C.

### 3.8. RT-PCR

Primers were purchased from Hokkaido System Science (Hokkaido, Japan). Murine FASN, fibroblast growth factor 21 (FGF21), collagen 1A1 (COL1A1), Liver X Receptor alpha (LXR alpha), ATP-binding cassette, sub-family A, member 1 (Abca1), and Glucose 6 Phosphatase (G6P) were examined. GAPDH was used as an endogenous control. RT-PCR was performed using SYBR-Green real-time PCR Master Mix-Plus (Toyobo, Osaka, Japan) and an Applied Biosystems 7300 real-time PCR system (Applied Biosystems, Foster City, CA, USA) as recommended by the manufacturers. Primers are listed on Table 2.

**Table 2:** Primers used for real-time PCR.

Genes	Forward	Reverse	Ref.
FASN	5ʹ-GGAGGTGGTGATAGCCGGTAT-3ʹ	5ʹ-TGGGTAATCCATAGAGCCCAG-3ʹ	[19]
FGF21	5ʹ-CTGCTGGGGGTCTACCAAG-3ʹ	5ʹ-CTGCGCCTACCACTGTTCC-3ʹ	[19]
COL1A1	5ʹ-TTCAGCTTTGTGGACCTCCG-3ʹ	5ʹ-TTGCACGTCATCGCACACAG-3ʹ	[35]
LXR alpha	5ʹ-CTCAATGCCTGATGTTTCTCCT-3ʹ	5ʹ-TCCAACCCTATCCCTAAAGCAA-3ʹ	[19]
Abca 1	5ʹ-GCTTGTTGGCCTCAGTTAAGG-3ʹ	5ʹ-GTAGCTCAGGCGTACAGAGAT-3ʹ	[36]
G6P	5ʹ-CGACTCGCTATCTCCAAGTGA-3ʹ	5ʹ-GTTGAACCAGTCTCCGACCA-3ʹ	[37]
GAPDH	5ʹ-TGCATCCTGCACCACCAACT-3ʹ	5ʹ-AACACGGAAGGCCATGCCAG-3ʹ	[38]

### 3.9. Statistical Analysis

All data are expressed as the mean ± SD of samples. Comparisons between the two groups were made using Mann-Whitney’s *U* test. In all cases, a *p*-value <0.05 was considered significant.

## 4. Conclusions

In conclusion, these findings demonstrate that 1,8-cineole might exert its hepatoprotective activity by reducing steatosis and fibrosis in Pten KO mice *in vitro* and *in vivo*. 1,8-cineole shows promise as a strong and safe therapeutic agent for NASH.

## References

[1] Cohen J.C., Horton J.D., Hobbs H.H. (2011). Human fatty liver disease: old questions and new insights. Science.

[2] Duvnjak M., Lerotić I., Baršić N., Tomašić V., Virović Jukić L., Velagić V. (2007). Pathogenesis and management issues for non-alcoholic fatty liver disease. World J. Gastroenterol..

[3] Day C.P., James O.F. (1998). Steatohepatitis: A tale of two ‘‘hits’’?. Gastroenterology.

[4] Qiu W., Federico L., Naples M., Avramoglu R.K., Meshkani R., Zhang J., Tsai J., Hussain M., Dai K., Iqbal J. (2008). Phosphatase and tensin homolog (PTEN) regulates hepatic lipogenesis, microsomal triglyceride transfer protein, and the secretion of apolipoprotein B–containing lipoproteins. Hepatology.

[5] Horie Y., Suzuki A., Kataoka E., Sasaki T., Hamada K., Sasaki J., Mizuno K., Hasegawa G., Kishimoto H., Iizuka M. (2004). Hepatocyte-specific Pten deficiency results in steatohepatitis and hepatocellular carcinomas. J. Clin. Investig..

[6] Giamakis A., Kretsi O., Chinou I., Spyropoulos C.G. (2001). *Eucalyptus camaldulensis*: Volatiles from immature flowers and high production of 1,8-cineole and beta-pinene by *in vitro* cultures. Phytochemistry.

[7] Tsiri D.O., Kretsi O., Chinou I.B., Spyropoulos C.G. (2003). Composition of fruit volatiles and annual changes in the volatiles of leaves of *Eucalyptus camandulensis* Dehn. Growing in Greece. Flavour Fragr. J..

[8] Levinson K.K., Takayama K., Isowa K., Okabe K., Nagai T. (1994). Formulation optimization of indomethacin gels containing a combination of three kinds of cyclic monoterpenes as percutaneous penetration enhancers. J. Pharm. Sci..

[9] Juergens U.R., Stöber M., Schmidt-Schilling L., Kleuver T., Vetter H. (1998). Anti-inflammatory effects of encalyptol (1,8-cineole) in bronchial asthma: inhibition of arachidonic acid metabolism in human blood monocytes *ex vivo*. Eur. J. Med. Res..

[10] Juergens U.R., Stöber M., Vetter H. (1998). Inhibition of cytokine production and arachidonic acid metabolism by eucalyptol (1,8-cineole) in human blood monocytes *in vitro*. Eur. J. Med. Res..

[11] Lahlou S., Figueieredo A.F., Magalhães P.J., Leal-Cardoso J.H. (2002). Cardiovascular effects of 1,8-cineole, a terpenoid oxide present in many plant essential oils, in normotensive rats. Can. J. Physiol. Pharmacol..

[12] Murata S., Shiragami R., Kosugi C., Tezuka T., Yamazaki M., Hirano A., Yoshimura Y., Suzuki M., Shuto K., Ohkohchi N. (2013). Antitumor effect of 1,8-cineole against colon cancer. Oncol. Rep..

[13] Cho K.H. (2012). 1,8-cineole protected human lipoproteins from modification by oxidation and glycation and exhibited serum lipid-lowering and anti-inflammatory activity in zebrafish. BMB Rep..

[14] Calvisi D.F., Wang C., Ho C., Ladu S., Lee S.A., Mattu S., Destefanis G., Delogu S., Zimmermann A., Ericsson J. (2011). Increased lipogenesis, induced by AKT-mTORC1-RPS6 signaling, promotes development of human hepatocellular carcinoma. Gastroenterology.

[15] Pal A., Barber T.M., de Bunt M.V., Rudge S.A., Zhang Q., Lachlan K.L., Cooper N.S., Linden H., Levy J.C., Wakelam M.J.O. (2012). *PTEN* mutations as a cause of constitutive insulin sensitivity and obesity. N. Eng. J. Med..

[16] Kuo Y.C., Huang K.Y., Yang C.H., Yang Y.S., Lee W.Y., Chiang C.W. (2008). Regulation of phosphorylation of Thr-308 of Akt, cell proliferation, and survival by the B55α regulatory subunit targeting of the protein phosphatase 2A holoenzyme to akt. J. Biol. Chem..

[17] Pommier A.J., Alves G., Viennois E., Bernard S., Communal Y., Sion B., Marceau G., Damon C., Mouzat K., Caira F. (2010). Liver X Receptor activation downregulates AKT survival signaling in lipid rafts and induces apoptosis of prostate cancer cells. Oncogene.

[18] Bradley M.N., Hong C., Chen M., Joseph S.B., Wilpitz D.C., Wang X., Lusis A.J., Collins A., Hseuh W.A., Collins J.L. (2007). Ligand activation of LXR beta reverses atherosclerosis and cellular cholesterol overload in mice lacking LXR alpha and apoE. J. Clin. Investig..

[19] Gao M., Bu L., Ma Y., Liu D. (2013). Concurrent activation of liver X receptor and peroxisome proliferator-activated receptor alpha exacerbates hepatic steatosis in high fat diet-induced obese mice. PLoS ONE.

[20] Jun H.J., Hoang M.H., Yeo S.K., Jia Y., Lee S.J. (2013). Induction of ABCA1 and ABCG1 expression by the liver X receptor modulator cineole in macrophages. Bioorg. Med. Chem. Lett..

[21] Badman M.K., Pissios P., Kennedy A.R., Koukos G., Flier J.S., Maratos-Flier E. (2007). Hepatic fibroblast growth factor 21 is regulated by PPARalpha and is a key mediator of hepatic lipid metabolism in ketotic states. Cell Metab..

[22] Dasarathy S., Yang Y., McCullougha A.J., Marczewski S., Bennet C., Kalhan S.C. (2011). Elevated hepatic fatty acid oxidation, high plasma fibroblast growth factor 21, and fasting bile acids in nonalcoholic steatohepatitis. Eur. J. Gastroenterol. Hepatol..

[23] Dushay J., Chui P.C., Gopalakrishnan G.S., Varela-Rey M., Crawley M., Fisher F.M., Badman M.K., Martinez-Chantar M.L., Maratos-Flier E. (2010). Increased fibroblast growth factor 21 in obesity and nonalcoholic fatty liver disease. Gastroenterology.

[24] Izumi Y., Bina H.A., Ouchi N., Akasaki Y., Kharitonenkov A., Walsh K. (2008). FGF21 is an Akt-regulated myokine. FEBS Lett..

[25] Uebanso T., Taketani Y., Yamamoto H., Amo K., Tanaka S., Arai H., Takei Y., Masuda M., Yamanaka-Okumura H., Takeda E. (2012). Liver X receptor negatively regulates fibroblast growth factor 21 in the fatty liver induced by cholesterol-enriched diet. J. Nutr. Biochem..

[26] Huang J.F., Chuang Y.H., Dai C.Y., Yu M.L., Huang C.F., Hsiao P.J., Hsieh M.Y., Huang C.I., Yeh M.L., Yang J.F. (2011). Hepatic Akt expression correlates with advanced fibrosis in patients with chronic hepatitis C infection. Hepatol. Res..

[27] Santos F.A., Silva R.M., Tomé A.R., Rao V.S., Pompeu M.M., Teixeira M.J., de Freitas L.A., de Souza V.L. (2001). 1,8-cineole protects against liver failure in an in-vivo murine model of endotoxemic shock. J. Pharm. Pharmacol..

[28] Lima P.R., de Melo T.S., Carvalho K.M., de Oliveira Í.B., Arruda B.R., de Castro Brito G.A., Rao V.S., Santos F.A. (2013). 1,8-cineole (eucalyptol) ameliorates cerulein-induced acute pancreatitis via modulation of cytokines, oxidative stress and NF-κB activity in mice. Life Sci..

[29] Xu J., Hu Z.Q., Wang C., Yin Z.Q., Wei Q., Zhou L.J., Li L., Du Y.H., Jia R.Y., Li M. (2014). Acute and subacute toxicity study of 1,8-cineole in mice. Int. J. Clin. Exp. Pathol..

[30] Rašković A., Milanović I., Pavlović N., Ćebović T., Vukmirović S., Mikov M. (2014). Antioxidant activity of rosemary (*Rosmarinus officinalis* L.) essential oil and its hepatoprotective potential. BMC Complement. Altern. Med..

[31] Suzuki A., Yamaguchi M.T., Ohteki T., Sasaki T., Kaisho T., Kimura Y., Yoshida R., Wakeham A., Higuchi T., Fukumoto M. (2001). T cell-specific loss of Pten leads to defects in central and peripheral tolerance. Immunity.

[32] Postic C., Magnuson M.A. (2000). DNA excision in liver by an albumin-Cre transgene occurs progressively with age. Genesis.

[33] Ishii H., Horie Y., Ohshima S., Anezaki Y., Kinoshita N., Dohmen T., Kataoka E., Sato W., Goto T., Sasaki J. (2009). Eicosapentaenoic acid ameliorates steatohepatitis and hepatocellular carcinoma in hepatocyte-specific Pten-deficient mice. J. Hepatol..

[34] Abramoff M.D., Magalhães P.J., Ram S.J. (2004). Image processing with ImageJ. Biophotonics Int..

[35] Yata Y., Scanga A., Gillan A., Yang L., Reif S., Breindl M., Brenner D.A., Rippe R.A. (2003). DNase I–hypersensitive sites enhance a1pha (I) collagen gene expression in hepatic stellate cells. Hepatology.

[36] Gao M., Liu D. (2013). Resveratrol suppresses T0901317-induced hepatic fat accumulation in mice. AAPS J..

[37] Wang L.J., Zhang H.W., Zhou J.Y., Liu Y., Yang Y., Chen X.L., Zhu C.H., Zheng R.D., Ling W.H., Zhu H.L. (2014). Betaine attenuates hepatic steatosis by reducing methylation of the MTTP promoter and elevating genomic methylation in mice fed a high-fat diet. J. Nutr. Biochem..

[38] Zender L., Hütker S., Liedtke C., Tillmann H.L., Zender S., Mundt B., Waltemathe M., Gosling T., Flemming P., Malek N.P. (2003). Caspase 8 small interfering RNA prevents acute liver failure in mice. Proc. Natl. Acad. Sci. USA.

